# Supplementary material for: In situ-formable, dynamic crosslinked poly(ethylene glycol) carrier for localized adeno-associated virus infection and reduced off-target effects
Source: Commun Biol. 2023 May 16;6:508. doi: 10.1038/s42003-023-04851-w (PMC10188511; doi:10.1038/s42003-023-04851-w)
Supplement: Supplementary file 2 — Supplementary Information [file 42003_2023_4851_MOESM2_ESM.pdf]

In situ-formable, dynamic crosslinked poly(ethylene glycol) carrier for localized adeno-associated virus infection

Motoi Kato[1], Shohei Ishikawa[2], Shen Qi[1], Du Zening[1], Takuya Katashima[2], Mitsuru Naito[3], Takao Numahata[1], Mutsumi Okazaki[1], Takamasa Sakai[2], and Masakazu Kurita[1]

[1] Department of Plastic and Reconstructive Surgery, Graduate School of Medicine, The University of Tokyo, 7-3-1, Hongo, Bunkyo-ku, Tokyo, Japan. [2] Department of Material Bioengineering, School of Engineering, The University of Tokyo, 7-3-1, Hongo, Bunkyo-ku, Tokyo, Japan. [3] Center for Disease Biology and Integrative Medicine, Graduate School of Medicine, The University of Tokyo, 7-3-1, Hongo, Bunkyo-ku, Tokyo, Japan

Supplementary Figure 1 | Normalized fluorescence intensity after photobleaching for PEG slime and PEG hydrogel

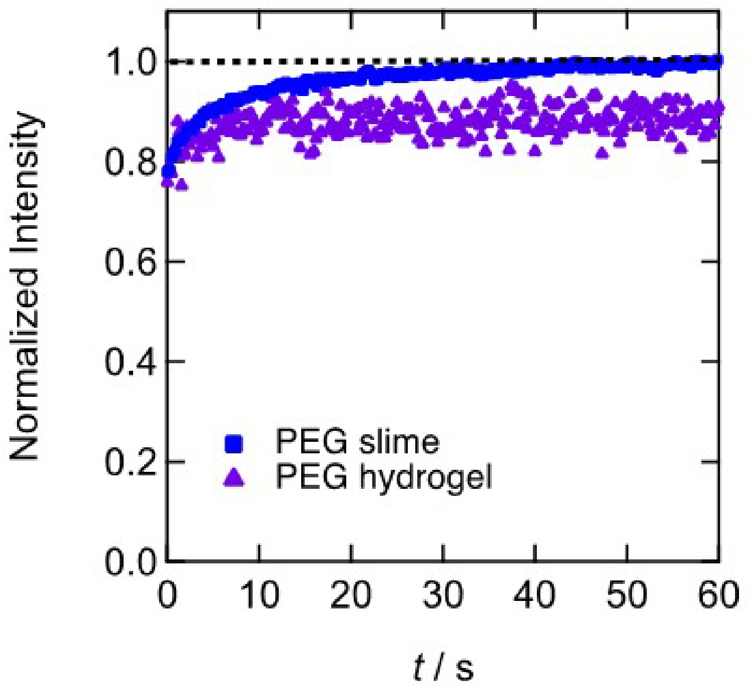

The fluorescence intensity of PEG slime was completely recovered approximately 60 s after photobleaching, whereas that of PEG gel was unchanged. This confirms the translational movement of the constitutive molecules of PEG slime and the dynamic crosslinking between these molecules. Blue square: PEG slime. Purple triangle: PEG hydrogel.

Supplementary Figure 2 | Skin ulcer model for comparative assessment of gene transduction efficiency.

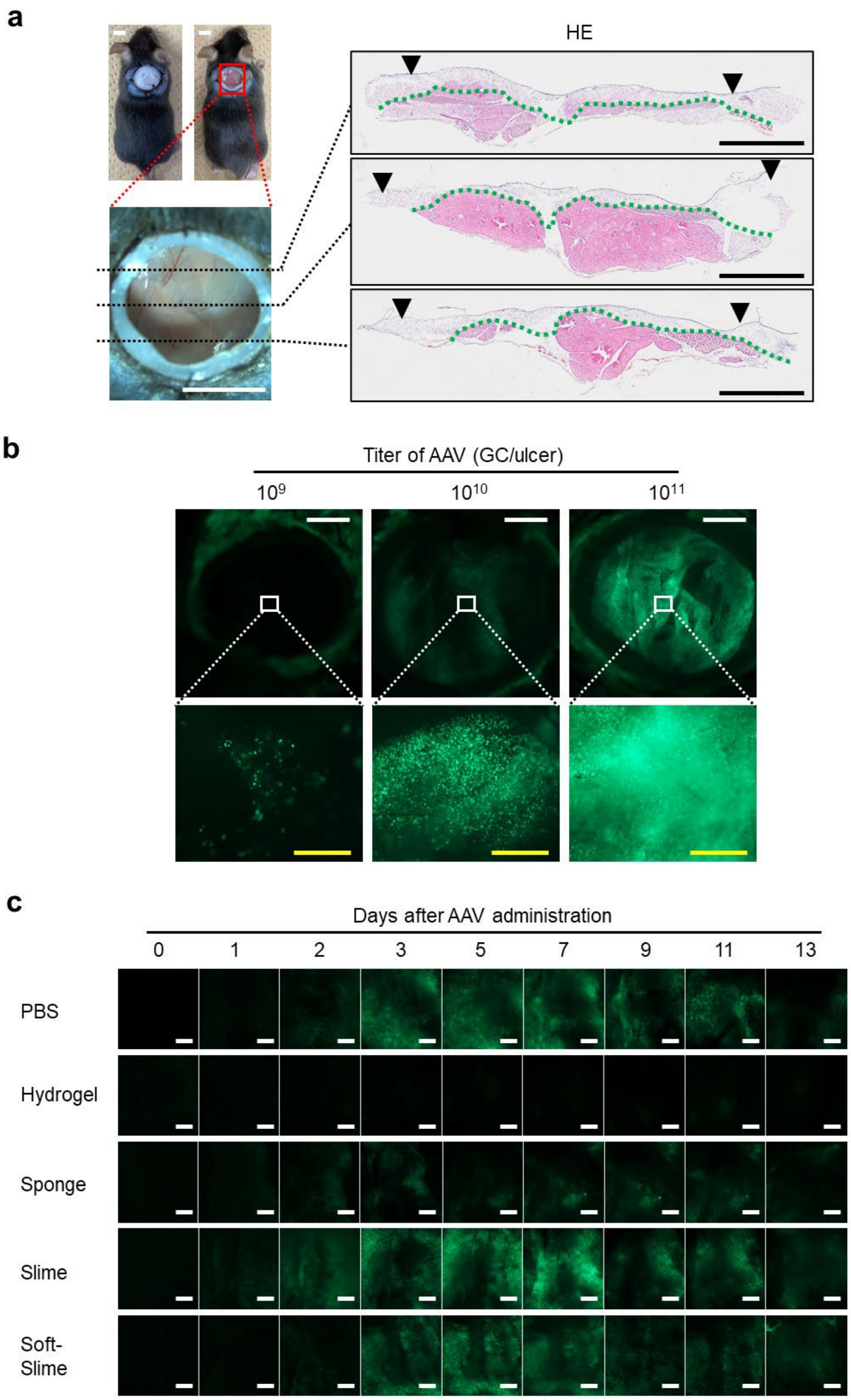

a, Appearance (left above) of skin ulcer with silicone chamber on the interscapular area before and after chopping off the roof of the chamber. Stereoscopic image of the chamber (left below) and H&E staining of sections (right) through the ulcer. Black dotted lines indicate the approximate position of sections. Arrow heads indicate the approximate position of edges observable in stereoscopic analyses. Green dotted lines indicate the boundary between superficial (granulation and adipose tissue) and deep layers (fascia and muscle). White scale bars = 5 mm; black scale bars = 2mm. **b**, Representative stereoscopic analysis of the whole chamber (above) and the center of the ulcer (below) 72 h after the administration of different titers of GFPNLS-AAVDJ. For quantitative comparison of positive cell numbers, 10^{10} GC per ulcer was appropriate. White scale bars = 2 mm; yellow scale bars = 300 μm. *n* = 3 for each titer, all similar results. **c**, Representative stereoscopic analysis of the center of the ulcer after the administration of GFPNLS-AAVDJ (10^{10} GC per ulcer) in different carriers. Images were obtained 0, 1, 2, 3, 5, 7, 9, 11, and 13 days after AAV administration under anesthesia. Relative GFP expression levels after day 3were consistent among different carriers. Bars = 1 mm. *n* = 3 for each carrier, all similar results.

Supplementary Figure 3 | Assessment of duplicated gene transduction efficiency

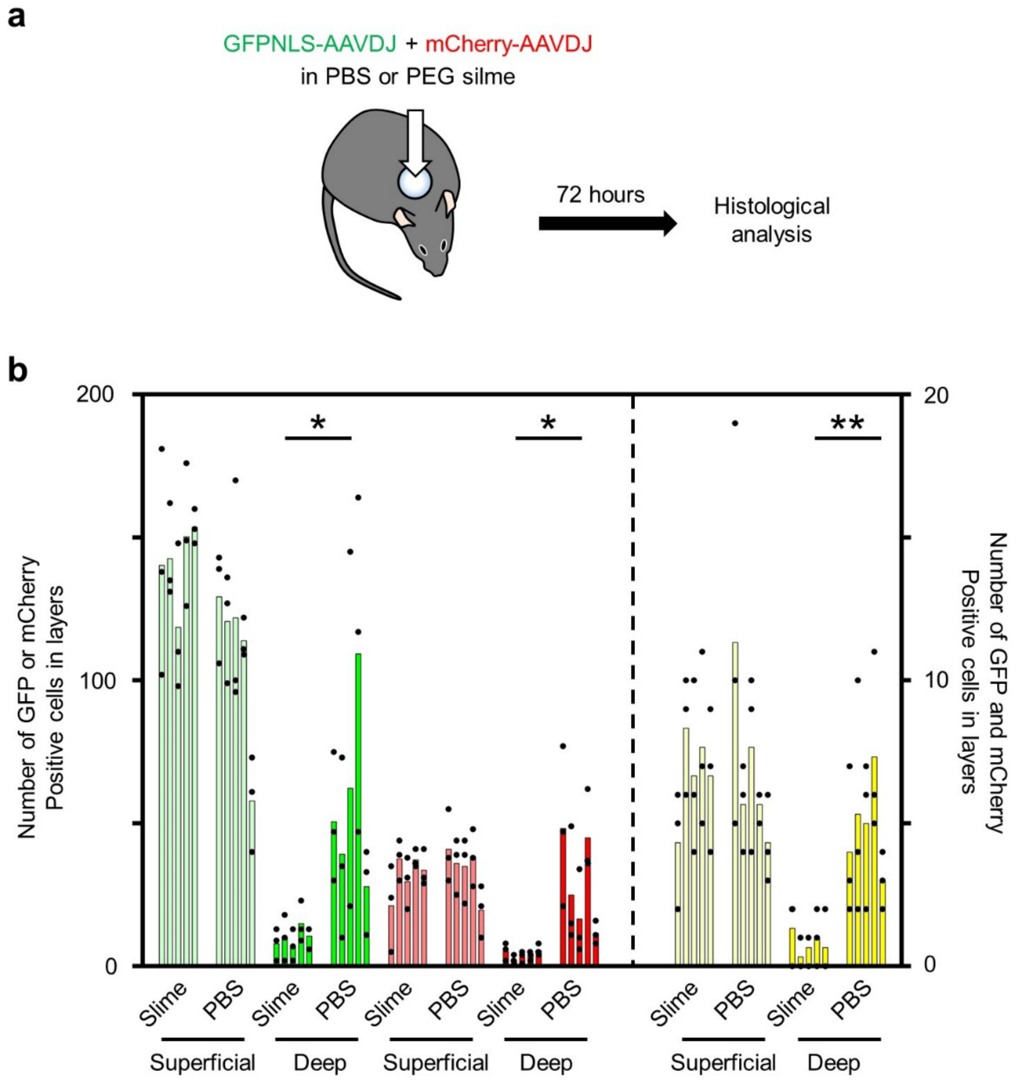

a, Mixture of GFPNLS-AAVDJ and mCherryNLS-AAVDJ (1× 10^{10} GC each) encapsulated in PEG slime or diluted with PBS was administered to a raw surface created on mice backs. **b,** Number of GFPNLS-positive cells and mCherryNLS-positive cells in the superficial and deep layers of each animal. The overlaid dot plot indicates the distribution of the data. The number of duplicated gene transduction in the superficial layer was consistent between PBS and PEG slime. * < 0.05, ** < 0.01 (*n* = 5).

Supplementary Figure 4 | Tracking of fluorescent labelled AAVs

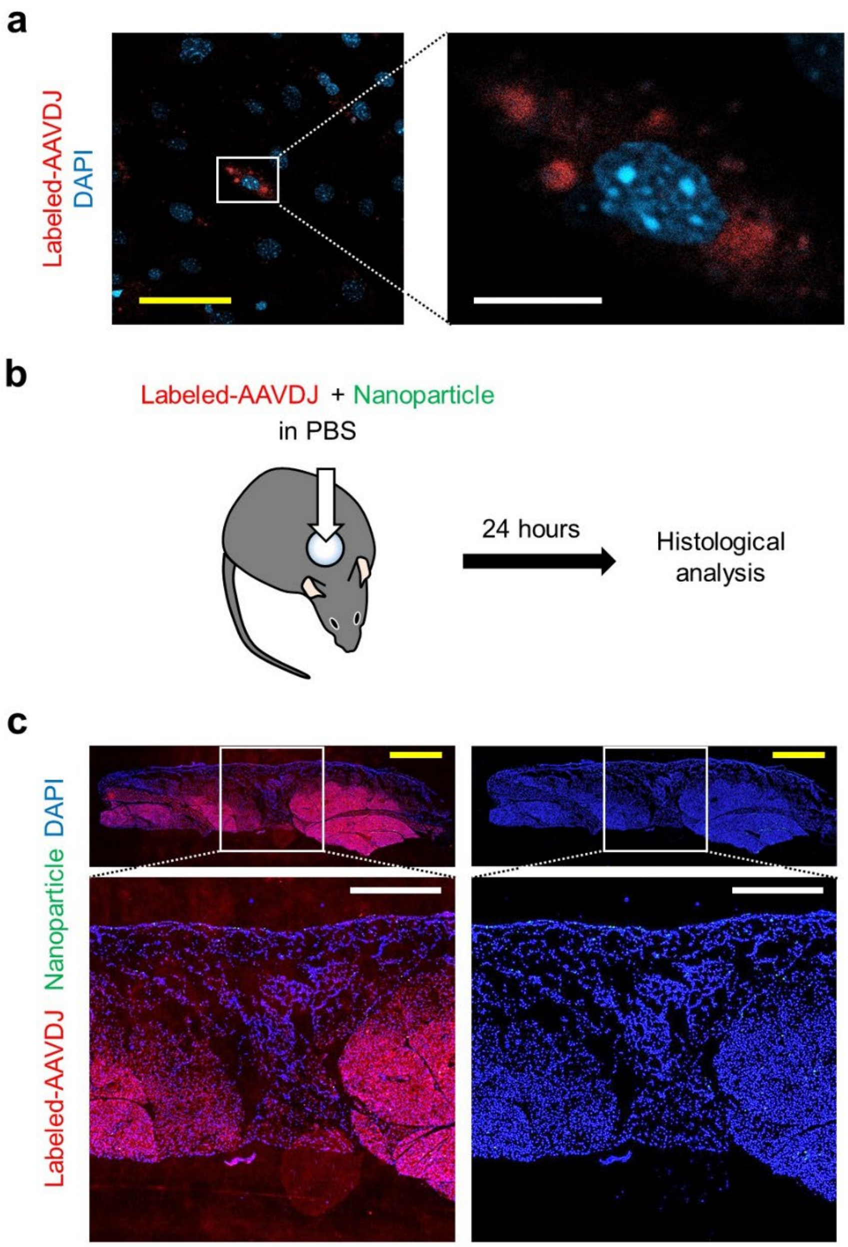

a, Primary mASCs incubated with Alexa Fluor® 568-labeled *DNP63A*-AAVDJ. Labeled AAVDJ could be tracked in cytosol *in vitro*. Yellow bar = 50 μm, White bar = 10 μm. **b,** Labeled AAVDJ and silica nanoparticle mixture was administered to a raw surface created on mice backs. Tissues were collected 24 h later and rendered for histological analyses (n = 3). **c,** Representative histological images. Reliable detection of labeled AAVDJ in the background was difficult, while the signal of each silica nanoparticle could be identified. Yellow bar = 500 μm, White bar = 1 mm.

Supplementary Figure 5 | Diffusion of fluorescent particles from the ulcer surface.

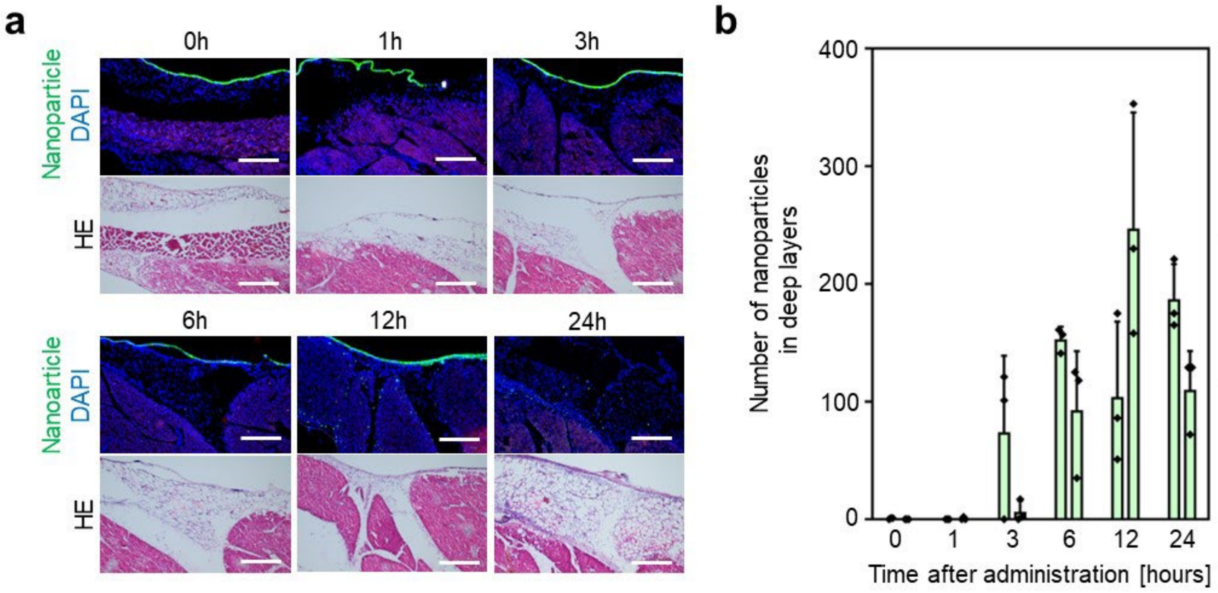

a, Representative slices of each time point after green-fluorescent silica nanoparticles suspended in PBS were applied in a chamber, set on a skin ulcer on the back of a mouse. Bar = 400 μm, h: hour(s) after administration. **b,** Number of nanoparticles in deep layers of each time point after administration. The overlaid dot plot indicates the distribution of the data. The particles were diffused into the deep layers of the skin over time. After 24 h of administration, most of the particles had disappeared from the surface of the ulcer.
