# Supplementary material for: In situ-formable, dynamic crosslinked poly(ethylene glycol) carrier for localized adeno-associated virus infection and reduced off-target effects
Source: Commun Biol. 2023 May 16;6:508. doi: 10.1038/s42003-023-04851-w (PMC10188511; doi:10.1038/s42003-023-04851-w)
Supplement: Supplementary file 5 — Reporting Summary [file 42003_2023_4851_MOESM5_ESM.pdf]

nature portfolio

Corresponding author(s): Masakazu Kurita

Last updated by author(s): Mar 10, 2023

Reporting Summary

Nature Portfolio wishes to improve the reproducibility of the work that we publish. This form provides structure for consistency and transparency in reporting. For further information on Nature Portfolio policies, see our Editorial Policies and the Editorial Policy Checklist.

Statistics

For all statistical analyses, confirm that the following items are present in the figure legend, table legend, main text, or Methods section.

n/a	Confirmed	
☐	☒	The exact sample size (n) for each experimental group/condition, given as a discrete number and unit of measurement
☐	☒	A statement on whether measurements were taken from distinct samples or whether the same sample was measured repeatedly
☐	☒	The statistical test(s) used AND whether they are one- or two-sided<br>*Only common tests should be described solely by name; describe more complex techniques in the Methods section.*
☒	☐	A description of all covariates tested
☒	☐	A description of any assumptions or corrections, such as tests of normality and adjustment for multiple comparisons
☒	☐	A full description of the statistical parameters including central tendency (e.g. means) or other basic estimates (e.g. regression coefficient) AND variation (e.g. standard deviation) or associated estimates of uncertainty (e.g. confidence intervals)
☒	☐	For null hypothesis testing, the test statistic (e.g. F, t, r) with confidence intervals, effect sizes, degrees of freedom and P value noted<br>*Give P values as exact values whenever suitable.*
☒	☐	For Bayesian analysis, information on the choice of priors and Markov chain Monte Carlo settings
☒	☐	For hierarchical and complex designs, identification of the appropriate level for tests and full reporting of outcomes
☒	☐	Estimates of effect sizes (e.g. Cohen's d, Pearson's r), indicating how they were calculated

Our web collection on statistics for biologists contains articles on many of the points above.

Software and code

Policy information about availability of computer code

Data collection	Olympus OlyVIA ver 3.3, Olympus VS 200 ASW V3.3, ZEISS ZEN pro
Data analysis	Image J bundled with Java 1.8.0, Excel 365

For manuscripts utilizing custom algorithms or software that are central to the research but not yet described in published literature, software must be made available to editors and reviewers. We strongly encourage code deposition in a community repository (e.g. GitHub). See the Nature Portfolio guidelines for submitting code & software for further information.

Data

Policy information about availability of data

All manuscripts must include a data availability statement. This statement should provide the following information, where applicable:

- Accession codes, unique identifiers, or web links for publicly available datasets
- A description of any restrictions on data availability
- For clinical datasets or third party data, please ensure that the statement adheres to our policy

All data generated or analysed during this study are included in this published article (and its supplementary information files).

Human research participants

Policy information about studies involving human research participants and Sex and Gender in Research.

Reporting on sex and gender	N/A
Population characteristics	N/A
Recruitment	N/A
Ethics oversight	N/A

Note that full information on the approval of the study protocol must also be provided in the manuscript.

Field-specific reporting

Please select the one below that is the best fit for your research. If you are not sure, read the appropriate sections before making your selection.

☒ Life sciences ☐ Behavioural & social sciences ☐ Ecological, evolutionary & environmental sciences

For a reference copy of the document with all sections, see nature.com/documents/nr-reporting-summary-flat.pdf

Life sciences study design

All studies must disclose on these points even when the disclosure is negative.

Sample size	No sample-size calculation was performed. For experiments on locus phenomenon assessments, the individual differences were considered to be small, the sample size was set to 3 in order to minimize the number of animals used. For experiments in which individual differences were expected, such as off-target hepatic infections, the sample size was set as 7 to avoid an extremely large number of animals.
Data exclusions	No data were excluded from the analyses.
Replication	The localization effect with using specific PEG on ulcer surface were confirmed as reproducible, due to the quite similar results from previous trials and the presented experiments.
Randomization	All the mice in the study were allocated randomly. In the chamber assay, the attachment of chamber was performed previously to the agents administration, with no labeling.
Blinding	The results were extracted by a single investigator among the first authors who mainly performed the experiments, and no special blinding was performed. However, since the manipulations were performed in a flow process, differences in drug administration and other manipulations were unlikely to occur, and the differences in results due to blinding were considered as little.

Reporting for specific materials, systems and methods

We require information from authors about some types of materials, experimental systems and methods used in many studies. Here, indicate whether each material, system or method listed is relevant to your study. If you are not sure if a list item applies to your research, read the appropriate section before selecting a response.

Materials & experimental systems

n/a	Involved in the study	
☒	☐	Antibodies
☒	☐	Eukaryotic cell lines
☒	☐	Palaeontology and archaeology
☐	☒	Animals and other organisms
☒	☐	Clinical data
☒	☐	Dual use research of concern

Methods

n/a	Involved in the study	
☒	☐	ChIP-seq
☒	☐	Flow cytometry
☒	☐	MRI-based neuroimaging

Animals and other research organisms

Policy information about studies involving animals; ARRIVE guidelines recommended for reporting animal research, and Sex and Gender in Research

Laboratory animals	C57BL/6JJcl, from Jackson, purchased from Nippon Bio-Supp. Center
Wild animals	The study did not include wild animals.
Reporting on sex	All animals used in the present study were female, because they were less aggressive in terms of mechanical damages on the surgically attached chambers when assessing with skin ulcer model.
Field-collected samples	The study did not involve samples collected from the fields.
Ethics oversight	All animal experiments were approved by the Animal Research Committee of the The University of Tokyo.

Note that full information on the approval of the study protocol must also be provided in the manuscript.
